# Guidelines on COVID-19 vaccination in patients with immune-mediated rheumatic diseases: a Brazilian Society of Rheumatology task force

**DOI:** 10.1186/s42358-022-00234-7

**Published:** 2022-01-17

**Authors:** Anna Carolina Faria Moreira Gomes Tavares, Ana Karla Guedes de Melo, Vítor Alves Cruz, Viviane Angelina de Souza, Joana Starling de Carvalho, Ketty Lysie Libardi Lira Machado, Lilian David de Azevedo Valadares, Edgard Torres dos Reis Neto, Rodrigo Poubel Vieira de Rezende, Maria Fernanda Brandão de Resende Guimarães, Gilda Aparecida Ferreira, Alessandra de Sousa Braz, Rejane Maria Rodrigues de Abreu Vieira, Marcelo de Medeiros Pinheiro, Sandra Lúcia Euzébio Ribeiro, Blanca Elena Gomes Rios Bica, Kátia Lino Baptista, Izaias Pereira da Costa, Claudia Diniz Lopes Marques, Maria Lúcia Lemos Lopes, José Eduardo Martinez, Rina Dalva Neubarth Giorgi, Lícia Maria Henrique da Mota, Marcos Antônio Araújo da Rocha Loures, Eduardo dos Santos Paiva, Odirlei André Monticielo, Ricardo Machado Xavier, Adriana Maria Kakehasi, Gecilmara Cristina Salviato Pileggi

**Affiliations:** 1 Hospital das Clínicas, Universidade Federal de Minas Gerais, Belo Horizonte, Brazil; 2 Hospital Universitário Lauro Wanderley, Universidade Federal da Paraíba, R. Tab. Stanislau Eloy, 585 - Castelo Branco, João Pessoa, Paraíba 58050-585 Brazil; 3 Hospital das Clínicas, Universidade Federal de Goiás, Goiânia, Brazil; 4 Universidade Federal de Juiz de Fora, Juiz de Fora, Brazil; 5 Hospital Universitário Cassiano Antônio Moraes, Universidade Federal do Espírito Santo, Vitória, Brazil; 6 Hospital Getúlio Vargas, Recife, Brazil; 7 Universidade Federal de São Paulo, São Paulo, Brazil; 8 Universidade Federal Fluminense, Niterói, Brazil; 9 Hospital Geral, Fortaleza, Brasil; 10 Universidade Federal do Amazonas, Manaus, Brazil; 11 Universidade Federal do Rio de Janeiro, Rio de Janeiro, Brazil; 12 Faculdade de Medicina, Universidade Federal do Mato Grosso do Sul, Campo Grande, Brazil; 13 Universidade Federal de Pernambuco, Recife, Brazil; 14 Universidade Federal de Ciências da Saúde, Porto Alegre, Brazil; 15 Pontifícia Universidade Católica, São Paulo, Brazil; 16 Instituto de Assistência Médica ao Servidor Público Estadual, São Paulo, Brazil; 17 Hospital Universitário, Brasília, Brazil; 18 Universidade Estadual, Maringá, Brazil; 19 Hospital das Clínicas, Universidade Federal do Paraná, Curitiba, Brazil; 20 Hospital das Clínicas, Universidade Federal do Rio Grande do Sul, Porto Alegre, Brazil

**Keywords:** Covid-19 vaccination, Task force, Guidelines, Immune-mediated rheumatic diseases, Immunosuppression

## Abstract

**Objective:**

To provide guidelines on the coronavirus disease 2019 (COVID-19) vaccination in patients with immune-mediated rheumatic diseases (IMRD) to rheumatologists considering specific scenarios of the daily practice based on the shared-making decision (SMD) process.

**Methods:**

A task force was constituted by 24 rheumatologists (panel members), with clinical and research expertise in immunizations and infectious diseases in immunocompromised patients, endorsed by the Brazilian Society of Rheumatology (BSR), to develop guidelines for COVID-19 vaccination in patients with IMRD. A consensus was built through the Delphi method and involved four rounds of anonymous voting, where five options were used to determine the level of agreement (LOA), based on the Likert Scale: (1) strongly disagree; (2) disagree, (3) neither agree nor disagree (neutral); (4) agree; and (5) strongly agree. Nineteen questions were addressed and discussed via teleconference to formulate the answers. In order to identify the relevant data on COVID-19 vaccines, a search with standardized descriptors and synonyms was performed on September 10th, 2021, of the MEDLINE, EMBASE, Cochrane Central Register of Controlled Trials, ClinicalTrials.gov, and LILACS to identify studies of interest. We used the Newcastle–Ottawa Scale to assess the quality of nonrandomized studies.

**Results:**

All the nineteen questions-answers (Q&A) were approved by the BSR Task Force with more than 80% of panelists voting options 4—agree—and 5—strongly agree—, and a consensus was reached. These Guidelines were focused in SMD on the most appropriate timing for IMRD patients to get vaccinated to reach the adequate covid-19 vaccination response.

**Conclusion:**

These guidelines were developed by a BSR Task Force with a high LOA among panelists, based on the literature review of published studies and expert opinion for COVID-19 vaccination in IMRD patients. Noteworthy, in the pandemic period, up to the time of the review and the consensus process for this document, high-quality evidence was scarce. Thus, it is not a substitute for clinical judgment.

**Supplementary Information:**

The online version contains supplementary material available at 10.1186/s42358-022-00234-7.

## Background

The pandemic of severe acute respiratory syndrome coronavirus-2 (SARS-CoV-2), whose first case was described in Wuhan, China, in December 2019 [1], is the most significant health crisis being faced by humanity currently, and this has motivated efforts by the scientific community to seek ways to combat the transmission of this new virus. The virus has spread rapidly worldwide, with more than 228 million confirmed cases. In Brazil, it has resulted in more than 591,000 cumulative deaths, constituting one of the largest, if not the greatest, epidemiological tragedies in our history [2, 3].

Nowadays, four vaccines against SARS-CoV-2 are approved for use in Brazil: CoronaVac/Butantan Institute—inactivated –, ChAdOX1 nCoV19/Oxford/AstraZeneca/Oswaldo Cruz Foundation—viral vector—, BNT162b2mRNA/Pfizer—messenger RNA—and Janssen Vaccine—viral vector.

Although reports from several cohorts of patients with IMRD published around the world do not show an increased risk of unfavorable outcomes associated with coronavirus disease 2019 (COVID-19) compared to the general population [4], new data from population registries [5], including the Brazilian Registry of IMRD patients infected by the SARS-CoV-2 named ReumaCov, showed that patients with a moderate to a high degree of immunosuppression and those with an uncontrolled disease, especially systemic lupus erythematosus, systemic vasculitis and systemic sclerosis with pulmonary involvement, are at increased risk for COVID-19 outcomes or death (pulse therapy with methylprednisolone or cyclophosphamide [prevalence ratio, PR 2.86; 95% CI 1.59 to 5.14; *p* < 0.018]) [6].

Vaccination is the best way to avoid immune-preventable infectious diseases. Patients with IMRD may have a reduced immune response due to the underlying disease or immunosuppressive treatment. Therefore, they have a higher risk of infections, the leading causes of hospitalizations and deaths in this group of patients [7]. For this reason, the discussion on immunization against SARS-CoV-2 has become an urgent and relevant issue [8]. The concern is shared about the effectiveness of different vaccines in patients with IMRD due to the disease itself and immunosuppressive drugs. Regarding the vaccine response, it is essential to emphasize that cellular and humoral immune responses are essential, and isolated response measures can lead to a mistaken idea of ineffectiveness [7].

Considering the uncertainties and the scarcity of data on the safety and efficacy of COVID-19 vaccination in patients with IMRD, the BSR Committee for Infectious and Endemic Diseases formed a task force composed of 24 rheumatologists to reach a consensus on COVID-19 vaccination using the Delphi method. This task force produced a consensus-based practical framework for COVID-19 vaccination in patients with IMRD. These recommendations are not intended to replace clinical judgment, and the vaccination decision should be individualized and shared between patients and rheumatologists.

## Methods

### Taskforce

The BSR first formed the task force with a steering committee that included the endemic and infectious diseases committee members and the executive board of the BSR. The task force was convened until July to August 2021 and comprised twenty-four rheumatologists: twenty rheumatologists specialized in treating diseases in adults and four pediatric rheumatologists from the BSR. The coordinator group, composed of nine rheumatologists, formulated a list of potential questions and a literature review. All nineteen concept questions were discussed and refined during two rounds of anonymous voting with other task force members. After questions approval, all  nineteen  answers were formulated by the coordinator group and then discussed in one round (3rd) with the whole group. In the last round (4th), all nineteen Q&A were voted. Each round consisted of the completion of a structured questionnaire to achieve a consensus.

### Literature review

The research questions were defined according to the efficacy, immunogenicity, and safety of available COVID-19 vaccines in adult IMRD patients and the influence of immunosuppressive therapy on vaccine immunogenicity.

**Fig. 1:**
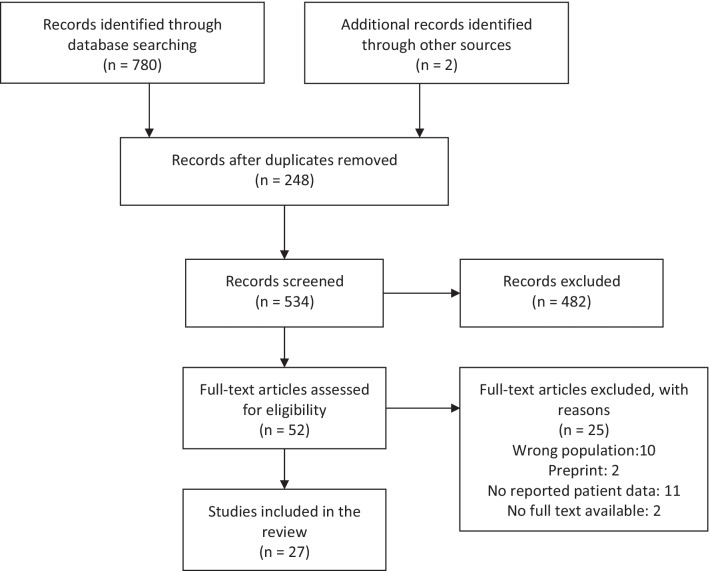
Study flow diagram

We searched MEDLINE, EMBASE, Cochrane Central Register of Controlled Trials (CENTRAL), and LILACS, using relevant descriptors and synonyms, adapting the search to the specifics of each database (Fig. 1). We also searched the Open Grey database, the World Health Organization International Clinical Trials Registry Platform (WHO ICTRP), and ClinicalTrials.gov to identify published, ongoing, and unpublished studies. All studies published before September 10th, 2021, were included, and no language restrictions were implemented for electronic search. Other papers that were considered relevant in the opinion of the experts could be added.

The search descriptors used for the Pubmed database were: "Rheumatic Diseases"[Mesh] OR "Autoimmune Diseases"[Mesh] OR "Connective Tissue Diseases"[Mesh] OR "Collagen Diseases"[Mesh] OR "Hereditary Autoinflammatory Diseases"[Mesh] OR (chronic immune inflammatory diseases) OR (immune-mediated inflammatory diseases) AND "COVID-19 Vaccines"[Mesh] OR "mRNA-1273 vaccine" [Supplementary Concept] OR "ChAdOx1 COVID-19 vaccine" [Supplementary Concept] OR "Ad5-nCoV vaccine" [Supplementary Concept] OR "Ad26.COV2.S vaccine" [Supplementary Concept] OR "BNT162 vaccine" [Supplementary Concept]. The search descriptors were adapted for each database used in this literature review.

The database search yielded 780 records. We also included two additional records. After removing duplicates, titles and abstracts were examined. We retrieved 52 full-text articles for further scrutiny; of those, 25 studies were excluded due to: ineligible population (n = 10), preprint (n = 2), not reporting patient data (n = 11), or no full text available (n = 2) (Fig. 1). We finally included 27 studies in this review [9–30].

Studies were eligible if they presented data on the immunogenicity, effectiveness, and/or safety of the COVID-19 vaccine in patients with IMRD. We used The Newcastle–Ottawa Scale (NOS) [31] (Additional file 1) to assess the methodological quality of the included studies.

### Consensus building

The entire process was conducted following the Delphi method, a well-established method for consensus-building [32, 33], and included four rounds of anonymous voting. Each round consisted of voting a structured online questionnaire built on the Google® Forms platform, and task force members received the questionnaire by e-mail. Based on the Likert Scale, all members should indicate their LOA for question–answer: 1. Strongly disagree; 2. Disagree, 3. Neither agree nor disagree (neutral); 4. Agree; and 5. Strongly agree. The first round consisted in discussing all questions with all members justifying agreement or disagreement to the questions. In the second round, the convener and collaborators made modifications considered plausible. All questions were voted according to the LOA mentioned above. In the third round, questions and answers were voted, and task force members had to justify agreement or disagreement for each Q&A; in the last round (4th), the coordinator group applied suggested modifications, and task force members voted statements-answers according to the LOA. More than 80% of panelists voted for options 4—agree—and 5—strongly agree in all scenarios, and the consensus was achieved, considering the LOA previously established by the coordinator group and following Delphi methodology. The manuscript was drafted and revised by all the task force members, and seventeen recommendations (Table 1) were established.

**Table 1:** Recommendations related to COVID-19 vaccination in patients with immune-mediated rheumatic diseases

Recommendations	LOA
1. Based on their risk for COVID-19, patients with IMRD should be encouraged to get their COVID-19 vaccination in a shared-making decision process	100%
2. The decision on the best timing to be vaccinated with COVID-19 vaccines should be individualized, considering the patient's age, the underlying IMRD, and its treatment, aiming to optimize the vaccine response	100%
3. COVID-19 vaccination should ideally occur in the setting of stable disease activity in patients with IMRD and absence or low immunosuppression	95.9%
4. The rheumatologist should inform their patients on the possibility of not effective vaccine response, especially those under high immunosuppression	100%
5. Immunomodulatory or immunosuppressive treatment in patients with IMRD should not be discontinued before and or after receiving COVID-19 vaccines, except for B-cell depleting agents (e.g., rituximab)	95.8%
6. COVID-19 vaccination should be ideally done 6 months after the last dose of rituximab and four weeks before the next one considering the complete vaccination schedule *If this is not possible, this recommendation should be followed at least for the first dose	95.8%
7. IMRD patients should receive the same COVID-19 vaccine platform in the complete schedule *In cases of severe adverse events (anaphylaxis) or immediate reactions (urticaria, angioedema, or respiratory distress) to any vaccine platform, an alternative approach is recommended for additional doses following local availability	95.8%
8. An additional dose of the COVID-19 vaccine should be considered for patients with IMRD who completed their vaccination schedule	100%
9. The additional dose should preferably be with a COVID-19 vaccine platform different than that used in the primary COVID-19 vaccination schedule	95.8%
10. Temporary interruption of immunomodulatory drugs before an additional dose of COVID-19 vaccines for patients with IMRD should not be recommended	91.7%
11. Temporary interruption of rituximab should not be recommended concerning the COVID-19 vaccine additional dose *Until high-quality evidence is available	83.4%
12. COVID-19 vaccines can be administered simultaneously with the other vaccines	92.3%
13. Assessment for seroconversion after COVID-19 vaccination is not recommended	95.9%
14. Seasonal influenza and pneumococcus vaccination are strongly recommended for patients with IMRD * It is essential to keep vaccination cards updated	92.3%
15. Vector viral COVID-19 vaccine should be recommended for patients with IMRD and thrombocytopenia or previous thrombotic events	95.9%
16. Pregnant patients with IMRD should receive only non-vector viral COVID-19 vaccines *Until new safety evidence is available for this scenario	100%
17. Children and adolescents (12–17 years) with IMRD should receive COVID-19 vaccination	95.8%

IMRD: immune-mediated rheumatic diseases

### Recommendations

#### Questions to explore

1. Should patients with IMRD receive COVID-19 vaccines?

Yes. Rheumatologists should be familiar with and be up to date on the characteristics, efficacy, and safety of COVID-19 vaccines to better guide their patients, considering both the local epidemiological situation and the risks and benefits of this SMD process.

LOA: 95.8% Strongly agree; 4.2% Agree

2. Should the decision on the best timing to get COVID-19 vaccination for patients with IMRD be individualized and preferably shared with the rheumatologist, considering the patient's age, the underlying IMRD, and its treatment, aiming to optimize the vaccine response?

Yes. Some factors, such as old age and comorbidities (mainly heart disease and chronic lung disease), are associated with unfavorable outcomes and a worse prognosis of COVID-19. The attending physician should consider IMRD activity and the degree of immunosuppression to determine the most appropriate time for COVID-19 vaccination in this population. In addition, rheumatologists should determine whether the patient fits the risk group for priority vaccination, as defined by the Public Health Agencies.

LOA: 83.3% Strongly agree; 16.7% Agree

3. Should the rheumatologist discuss with their patients the possibility of lower vaccine response, especially those under a high degree of immunosuppression?

Yes. Despite the scarcity of studies and the significant variability of immunosuppressants used in clinical practice, recent evidence suggests that patients under intense immunosuppression may have a lower vaccine response. Particular attention should be given to patients using doses greater than 20 mg/day of glucocorticoids and those using abatacept or rituximab due to their negative impact on vaccine response [34–37].

LOA: 83.3% Strongly agree; 16.7% Agree

4. Should the IDEAL timing get COVID-19 vaccination in patients with IMRD be when in remission or absence or low degree of immunosuppression?

Yes. Aiming for better vaccine response, IMRD patients should be vaccinated when in remission or under control with a low degree of immunosuppression or without immunosuppressive treatment [38–40]. In other situations, it is worth discussing with the attending rheumatologist on the best timing for vaccination, considering the epidemiological situation and the inclusion in the priority groups defined by the Ministry of Health and the associated factors specific to IMRD that are described above.

LOA: 79.2% Strongly Agree; 16.7% Agree

5. Based on the available literature data, should immunomodulatory/immunosuppressive treatment in patients with IMRD not be discontinued before and or after receiving COVID-19 vaccines? Should COVID-19 vaccination in IMRD patients be postponed while under treatment with a B-cell depleting agent (e.g., rituximab)?

Yes. To date, there are no available data to guide the management of immunosuppressive therapy in the context of COVID-19 vaccination. All the currently approved vaccines are non-live, and there is no risk of vaccine-related infection. It is also necessary to consider the risk of reactivation of the underlying disease with the interruption of a specific treatment and the potential negative effect of using immunosuppressive medications on vaccine response. Two studies recently published addressed COVID-19 vaccination in patients with IMRD. Braun-Moscovici et al. conducted a phase IV, prospective open-label trial to assess the humoral response after two doses of mRNA COVID-19 vaccine in patients with IMRD treated with immunomodulatory drugs and the impact on IMRD activity [34]. After multivariate logistic regression analysis, rituximab (RTX) and abatacept were related to decreased humoral response to a vaccine. The authors concluded that most disease-modifying antirheumatic drugs (DMARDs), including methotrexate (MTX), biologics, and Janus Kinase (Jak) inhibitors, can be continued with BNT162b2mRNA/Pfizer COVID-19 vaccine. Delaying treatment with RTX, when possible, should be considered in individual cases. Mrak et al. [35] evaluated humoral and cellular immune responses in RTX treated patients following COVID-19 vaccination. Twenty-nine (39%) out of 74 RTX treated patients seroconverted with BNT162b2mRNA/Pfizer immunization. Twenty-six out of 45 patients (58%) had a detectable cellular response. These data suggest that vaccination can induce SARS-CoV-2 specific antibodies in RTX-treated patients regardless of humoral response and may lead to new vaccination strategies in patients treated with RTX. A Brazilian study evaluated the immunogenicity and safety of the CoronaVac inactivated vaccine in patients with IMRD in a phase IV trial [36]. Lower anti-SARS-CoV2 IgG seroconversion (70.4 versus 95.5%, *p* < 0,001) and neutralizing antibodies positivity (NAbs) (56.3 versus 79.3%, *p* < 0.001) in the IMRD group compared to the control group were evidenced. The use of MTX (OR 0.42; 95% CI 0.29–0.61, *p* < 0.001), mycophenolate mofetil (OR 0.15; 95% CI 0.09–0.24, *p* < 0.001), tumor necrosis factor-alpha inhibitors (OR 0.41; 95% CI 0.26–0.64, *p* < 0.001), abatacept (OR 0.24; 95% CI 0.13–0.46, *p* < 0.001) and RTX (OR 0.34; 95% CI 0.13–0.93, *p* = 0.036) were associated with lower seroconversion in patients with IMRD. MTX (OR 0.67, 95% CI 0.47–0.95, *p* = 0.024), mycophenolate mofetil (OR 0.33; 95% CI 0.21–0.53, *p* < 0.001) and RTX (OR 0.28; 95% CI 0.09–0.87, *p* = 0.028) were associated with the absence of neutralizing activity in patients with IMRD. Together, these data provided evidence of humoral and cellular immunogenicity in a short-term follow-up of IMRD patients vaccinated with mRNA and inactivated COVID-19 vaccines. Long-term data is required. COVID-19 vaccination should be ideally done 6 months after the last dose of RTX and four weeks before the next one considering the complete schedule. If this is not possible, this recommendation should be followed, at least for the first dose.

LOA: 50% Strongly Agree; 45.8% Agree

6. Should COVID-19 vaccination be recommended for patients with IMRD even if they have already been infected with this virus?

Yes. Regardless of the scarcity of evidence about acquired immunity duration due to SARS-CoV-2 infection and the formal recommendation for vaccination of recovered patients from the general population, we recommend following the local health agencies’ guidelines until new evidence or recommendations are available. Cases of reinfection in humans or infection with new virus variants have been reported a few months after initial infection, challenging the idea of long-lasting protective immunity [41–43]. In addition, it is crucial to consider the scenario of the emergence of new virus variants.

LOA: 87.5% Strongly agree; 8.3% Agree

7. Should all platforms of COVID-19 vaccines be considered potentially safe for vaccination of patients with IMRD?

Yes. None of the platforms used to produce vaccines contain live viruses. Recently published studies [34–37] that assessed efficacy and safety of vaccines in patients with IMRD showed a low incidence of adverse events, most of them being local and self-limited. Guidelines from local health agencies should be followed until new evidence are available.

LOA: 70.8% Strongly agree; 29.2% Agree

8. Should the choice of COVID-19 vaccine for IMRD patients follow the recommendations of local regulatory agencies and availability?

Yes. The choice should follow the requirements of local public health agencies and the local availability of the vaccines. We recommend that all individuals, including patients with IMRD, receive a COVID-19 vaccine that has undergone a rigorous national regulatory process and is approved.

LOA: 83.3% Strongly agree; 16.7% Agree

9. Should IMRD patients receive the same platform in the complete schedule of COVID-19 vaccination until new evidence about interchangeability is available?

Yes. The entire vaccination schedule must follow the same platform initially used and should follow the national and local health regulatory agencies’ guidelines. In cases of severe adverse events (anaphylaxis) or immediate reactions (urticaria, angioedema, or respiratory distress) to any vaccine platform [44], an alternative approach is recommended for additional doses following local availability. Recently the National Immunization Program (NIP) recommended an additional dose of COVID-19 vaccine for immunocompromised patients, whose platform choice should be guided by local availability but should be different from the platform of the initial schedule [45].

LOA: 62.5% Strongly agree; 33.3% Agree

10. Should an additional dose of the COVID-19 vaccine be considered for patients with IMRD who completed their vaccination schedule?

Yes. To date, studies indicated that individuals with moderately to severely compromised immune systems may not build the same level of immunity to a 2-dose vaccine schedule compared to people who are not immunocompromised [32–35]. Therefore, they may benefit from an additional dose to ensure better protection against COVID-19. In addition, data on breakthrough infections have accounted for a large proportion of the hospitalization rate from fully vaccinated immunocompromised subjects [46].

LOA: 83.3% Strongly agree; 16.7% Agree

11. Should the additional dose preferably be with a COVID-19 vaccine platform different than that used in the primary COVID-19 vaccination schedule?

Yes. Available data on the responses to heterologous COVID-19 vaccination, especially in countries using inactivated and vector viral vaccines, showed higher spike RBD-IgG (receptor binding domain) and neutralizing activities than the homologous vaccine recipients [47–49].

LOA: 62.5% Strongly agree; 33.3% Agree

12. Should a temporary interruption of immunomodulatory drugs be recommended before an additional dose of COVID-19 vaccines for patients with IMRD?

No. Long-term data are required to guide recommendations for immunomodulatory/immunosuppressive drugs interruption and COVID-19 vaccination. So far, the literature data is conflicting and does not allow to take a definitive position.

LOA: 37.5% Strongly agree; 54.2% Agree

13. Should rituximab be interrupted concerning COVID-19 vaccine additional dose?

No. Regarding rituximab or other anti-CD20 therapies, the best period for vaccination must be discussed through an SMD process between the patient and assistant rheumatologist. Assessing serum CD19 levels [40] could be a tool to guide the best timing for the additional dose of the vaccine and subsequent dose of rituximab. If this is not available, it is recommended to perform the additional dose 2 to 4 weeks before the next dose of RTX. The available literature points out a possible influence of rituximab in seroconversion [32–36, 50], but more information is required.

LOA: 29.2% Strongly agree; 54.2% Agree

14. Is there a risk that the underlying disease will worsen or reactivate after COVID-19 vaccination?

Yes. Natural viral infection or vaccination have been noted as potential triggering events for inflammatory diseases for many decades. Regarding disease activity of IMRD and vaccination, it is suggested that promoting vaccination in quiescent disease phases avoids disease flare-up and favors a better immune response. However, due to COVID-19 severity, a rapid COVID-19 immunization is strongly recommended, and the patients included in most trials were vaccinated in different activity phases of their disease. Concerning the possibility of inducing or enhancing the autoimmune response through molecular mimicry between the viral antigen and host antigen, Rotondo et al. showed a low rate (5.7%) of disease relapse of IMRD after the first dose of vaccine, the rate of disease flare-up observed after BNT162b2mRNA/Pfizer vaccination could be due to the higher frequency of this vaccine administration. However, no significant differences in adverse events (AEs) between BNT162b2mRNA/Pfizer and ChAdOx1 nCoV19 were found in this study [51]. Watad et al. reported that the average time between vaccination and new-onset or flare of symptoms was four days (median of 4 days [1–25 days] in those who developed an IMRD after the first dose and a median of 4 days [1–7 days] in those after the second dose) with most cases occurring after the first inoculation (77.8%) [52]. Despite the high population exposure in the regions served by these centers, the authors concluded that IMRDs flares or onset temporally associated with COVID-19 vaccination appear rare. Most are moderate events and respond to therapy; although some severe flares occurred, new studies could be necessary.

LOA: 66.7% Strongly agree; 29.2% Agree

15. Is it recommended to assess for seroconversion after COVID-19 vaccination?

No. It is not recommended to assess the post-vaccination humoral response against SARS-CoV-2. The level of seroconversion varies among individuals, especially those immunocompromised. In addition, humoral immunity is not the only protective barrier against SARS-CoV-2, and the durability of the protection provided by natural infection and vaccination is not well-defined [35]. Individuals that received COVID-19 vaccines or those who recovered from infection should be informed that the durability of the protection is still to be determined.

LOA: 79.2% Strongly agree; 16.7% Agree

16. Should patients with IMRD also receive influenza and pneumococcus vaccines?

Yes. Vaccination against seasonal influenza and pneumococcus is strongly recommended for patients with IMRD, considering the risk of severe pneumonia in immunocompromised patients. COVID-19 vaccines can be administered with other vaccines at the same time, accordingly to National Immunization Program and the Ministry of Health [53]. We emphasize that it is important to keep IMRD patients vaccination card updated.

LOA: 73.1% strongly agree; 19.2% agree

17. Should vector viral vaccine be recommended for patients with IMRD and thrombocytopenia or previous thrombotic events?

Yes. Thromboembolic events related to viral vector vaccines are rare, with an average of 2 to 4 cases reported per million doses applied. Its immunological mechanism is described in immune-mediated thrombotic thrombocytopenia induced by heparin (HITT or HIT type 2), where thrombosis and thrombocytopenia are associated with anti-platelet factor IV antibodies. Although the risk factors for developing this adverse reaction are unknown, most cases were observed in women under 50 years old, without comorbidities or risk factors for thrombosis. There is no evidence of greater risk among IMRD patients and no plausible reason to contraindicate COVID-19 vaccination in this group. Vector viral vaccines should be avoided in individuals with a previous history of HITT and venous and or arterial thrombosis cases with thrombocytopenia after any vaccine [54–58].

LOA: 54.2% Strongly agree; 41.7% Agree

18. Should pregnant patients with IMRD receive only non-vector COVID-19 vaccines until new safety evidence is available for this scenario?

Yes. Vaccination should be encouraged in pregnant women [59], following guidelines of national health authorities. Since the notification of a fatal case of thrombosis with thrombocytopenia in a pregnant woman after the ChAdOx1- nCoV19 vaccine, the National Health Surveillance Agency and the Brazilian Ministry of Health discontinued the administration of vector viral vaccines in pregnant and postpartum women. Until such association is definitively clarified, Brazilian regulatory agencies recommended only m-RNA and inactivated virus COVID-19 vaccines for this group of individuals.

LOA: 58.3% Strongly agree; 41.7% Agree

19. Should children and adolescents (12–17 years old) with IMRD be vaccinated against SARS-CoV-2?

Yes. On June 23rd, 2021, the Center for Disease Control and Prevention reported an increased number of post-mRNA vaccine myocarditis/pericarditis cases (possibly hypersensitivity eosinophilic myocarditis). These are considered rare events, with no reported fatal outcomes. The majority of cases occurred in adolescents and young adults (under 30 years old), mainly boys over 12 to 17 years old. There are no long-term data on this outcome yet [60]. The BSR considers the benefits to outweigh the risks and recommends COVID-19 vaccination for this age group. LOA: 70.8% Strongly agree; 25% Agree.

## Discussion

This document presents the result of a BSR Task Force Delphi consensus-building to provide guidelines for COVID-19 vaccination in IMRD patients based on clinical scenarios. The consensus process considered potential concerns and was elaborated according to evidence-based information and expert opinion. The BSR developed these guidelines with a high LOA among their panelists regarding COVID-19 vaccination in patients with IMRD. These recommendations (Table 1) included discussion about particularities of safety and immunogenicity of vaccination in immunocompromised patients, considering adverse events and guidance for managing immunosuppressive treatment.

Vaccination is the most effective strategy to reduce and mitigate the SARS-CoV-2 pandemic. In randomized studies, COVID-19 vaccines proved efficacy in reducing SARS-CoV-2 infection rates and severe disease. However, the pivotal studies did not include IMRD patients who have an immune dysfunction related either to the underlying disease or the use of immune-modulating drugs, which could interfere in COVID-19 vaccination response. Both humoral and T-cell immune response following vaccination are relevant to evaluate effectiveness. Understanding of the development and durability of these responses determines the necessity for booster dosing schedules [34–37]. Currently, data have shown a lower response rate in IMRD patients when compared to healthy controls. It is unclear whether this is attributable to the underlying disease or its different treatments [38, 61].

The decision to vaccinate IMRD patients should be individualized and preferably shared between health professionals and patients, considering the epidemiological setting, the immunosuppression degree, and disease activity. Immunization should not be delayed in epidemics and pandemics such as COVID-19 since the benefits outweigh the risks. Misinformation negatively affects the decision to vaccinate, reduces the population's confidence, and decreases adherence to vaccination. SDM process should be based on highest scientific evidence and on persuasive and motivational approach strategies especially among pregnant women, children, adolescents and immunocompromised patients. This could be made discussing with patients risks and benefits of vaccines, listening to their doubts, clarifying their concerns and providing them ways to get high-quality information from confident sources. It seems to be the most suitable way to overcome barriers to vaccine hesitation, especially in vulnerable populations [62–65].

Vaccination hesitancy is a complex and multifactorial phenomenon defined as a delay in the acceptance of vaccination despite the availability of vaccination services. A study recently published by our group analyzed data from 1,000 patients with IMRD and evidenced a high vaccine acceptance rate (81.9%), but also found out that 25% of patients who hesitated to receive the COVID vaccine linked their decision to the lack of a definitive recommendation for vaccination against COVID-19, suggesting that physicians should be more engaged in disseminating information demystifying issues related to vaccination [64, 65].

All four COVID-19 vaccines approved in Brazil are non-live, and thus, there is no risk of inducing vaccine-related disease [51, 52]. Several studies confirmed that other non-live vaccines, such as those against influenza, pneumococcus, hepatitis A, hepatitis B, and human papillomavirus, are effective and safe for this population [66, 67]. Influenza and pneumococcus vaccinations are strongly recommended for IMRD patients because of an increased risk of developing severe pneumonia and its complications such as severe respiratory distress and respiratory failure [39, 66–69].

Some international Rheumatology Societies have released recommendations for COVID-19 vaccination in IMRD patients. The American College of Rheumatology (ACR) and the European Alliance of Associations for Rheumatology (EULAR) suggested that SMD process is essential in defining the best timing for vaccination and stand out the absence of safer and more effective vaccine platform for individuals with IMRD. The ACR pointed to temporary interruption of immunosuppressive agents before and/or after immunization, such as abatacept, MTX, and Jak inhibitors. This clinical guidance was based on observational studies assessing vaccination responses against other microorganisms, such as pneumococcus and influenza, and in the growing knowledge about the impact of immunosuppressants in COVID-19 vaccines responses. The use of new technologies, such as mRNA and viral vector-based, is relatively new, and the impact of the underlying disease and immune-modulating drugs on serological and T-cell responses are uncertain [7, 34–37]. The authors recognized that there is limited high-quality evidence to base these recommendations [39, 40].

Given this scenario of uncertainty, we performed a systematic literature review on the safety and efficacy of COVID-19 vaccines. Only non-randomized studies were found with heterogeneous subtypes of IMRD and diverse treatments regimens, absence of a well-defined control group, and differences in outcomes, leading to difficulties in interpreting the results and comparing the studies. We performed critical judgment of the included studies based on the selection of the study groups, the comparability of the groups, and the ascertainment of either the exposure or outcome using the Newcastle–Ottawa Scale.

Systematic reviews of prospective cohort studies could be the key to better understanding the effectiveness and safety of the COVID-19 vaccine in IMRD patients. We believe that the results of our review and recommendations may help decision-making processes and guide Brazilian rheumatologists in daily practice.

## Conclusion

The BSR task force approved a twenty Q&A with more than 80% LOA, considering scenarios of daily practice to help the decisions on COVID-19 vaccination in IMRD patients based on the SMD process and established seventeen recommendations (Table 1).

Although these guidelines are based on the best evidence available on the safety and immunogenicity of COVID-19 vaccines in patients with IMRD, we emphasize that there is still no high-quality evidence to guide the temporary withdrawal of immunomodulatory or immunosuppressive medications before or after COVID-19 vaccination and further research is required. It is noteworthy that the current consensus was built based on expert opinion and thus, do not intend to substitute clinical judgment. These Guidelines will be updated, As soon as new evidence about the COVID-19 vaccines’ safety and efficacy emerges.

## Supplementary Information

**Additional file 1.** Newcastle–Ottawa Quality Assessment Scales.

## Data Availability

Nor applicable.

## Abbreviations

ACR: American College of Rheumatology
BSR: Brazilian Society of Rheumatology
CG: Control group
COVID 19: Coronavirus disease 2019
DMARDs: Disease-modifying rheumatic drugs
EULAR: European Alliance of Associations for Rheumatology
IgG: Immunoglobulin G
IMRD: Immune-mediated rheumatic diseases
JAK: Janus kinase
LOA: Level of agreement
MTX: Methotrexate
mRNA: Ribonucleic acid messenger
NAb: Neutralizing antibody
NIP: National Immunization Programme
PR: Prevalence ratio
Q&A: Questions-answers
RBD: Receptor binding domain
ReumaCoV: Brazilian Registry of IMRD patients infected by the SARS-CoV-2
RTX: Rituximab
SARS-CoV-2: Severe acute respiratory syndrome coronavirus 2
SMD: Shared-making decision process
YF: Yellow fever
