# Supplementary material for: Guidelines on COVID-19 vaccination in patients with immune-mediated rheumatic diseases: a Brazilian Society of Rheumatology task force
Source: Adv Rheumatol. 2022 Jan 17;62(1):3. doi: 10.1186/s42358-022-00234-7 (PMC8762982; doi:10.1186/s42358-022-00234-7)
Supplement: Supplementary file 1 — Additional file 1. Newcastle–Ottawa Quality Assessment Scales. [file 42358_2022_234_MOESM1_ESM.docx]

**Table 1. NEWCASTLE-OTTAWA QUALITY ASSESSMENT SCALE CASE CONTROL STUDIES**

| **Study** | **Selection** | | | | **Comparability** | **Exposure** | | | **NOS*** |
| --- | --- | --- | --- | --- | --- | --- | --- | --- | --- |
|  | 1.Is the Case Definition Adequate? | 2.Representa  tiveness of the Cases | 3.Selection of Controls | 4.Definition of Controls | Comparability of cases and controls on the basis of the design or analysis | 1. Ascertain ment of exposure | 2. Same method of ascertainment for cases and controls | 3. Non-Response rate |  |
| Seyahi E, 2021 [9] | + | - | - | + | + | - | + | + | **5/9** |
| Rotondo C, 2021 [51] | + | + | - | + | + | - | + | - | **5/9** |
| Michiels Y, 2021 [10] | + | - | + | + | + | + | + | + | **7/9** |
| Veenstra J, 2021 [11] | + | + | + | - | + | + | + | - | **6/9** |
| Izmirly PM, 2021  [12] | + | + | + | + | + | + | + | - | **7/9** |
| Geisen UM, 2021 [50] | + | - | + | + | + | + | + | - | **6/9** |
| Prendecki M, 2021 [13] | + | + | - | - | + | + | + | - | **5/9** |
| Furer V, 2021 [37] | + | + | - | - | - | + | + | - | **5/9** |
| Cherian S, 2021 [14] | + | + | - | - | + | + | + | - | **5/9** |
| Cuomo G, 2021 [15] | - | - | - | - | + | - | + | - | **2/9** |
| Medeiros AC, 2021 [36] | + | + | - | - | + | + | + | - | **5/9** |

 *NOS= Newcastle-Ottawa Scale

**Table 2. NEWCASTLE-OTTAWA QUALITY ASSESSMENT SCALE**

**COHORT STUDIES**

| **Study** | **Selection** | | | | **Comparability** | **Outcome** | | |
| --- | --- | --- | --- | --- | --- | --- | --- | --- |
|  | 1. Representa   tiveness of the exposed cohort | 2. Selection of non-exposed cohort | 3. Ascertain ment of Exposure | 4. Demons tration that Outcome of Interest Was Not Present at Start of Study | Comparability of cohorts on the basis of the design or analysis | 1. Assess ment of outcome | 2. Was follow-up long enough for outcomes to occur | 3. Adequacy  of follow up  of cohorts |
| Chiang TP, 2021 [16] | + | - | + | + | - | + | + | - |

**Table 3. NEWCASTLE-OTTAWA QUALITY ASSESSMENT SCALE**

**CASE REPORTS AND CASE SERIES**

| **Study** | **Selection** | **Ascertainment** | | **Causality** | | | | **Reporting** |
| --- | --- | --- | --- | --- | --- | --- | --- | --- |
|  | 1) Does the patient(s) represent(s) the whole experience of the investigator (centre) or is the selection method unclear to the extent that other patients with similar presentation may not have been reported? | 2)Was the exposure adequately ascertained? | 3)Was the outcome adequately ascertained? | 4)Were other alternative causes that may explain the observation ruled out? | 5)Was there a challenge/rechallenge phenomenon? | 6)Was there a dose–response effect? | 7)Was follow-up long enough for outcomes to occur? | 8)Is the case(s) described with sufficient details to allow other investigators to replicate the research or to allow practitioners make inferences related to their own practice? |
| Benucci M, 2021 [17] | - | + | + | - | - | - | + | + |
| Niebel D, 2021 [18] | + | + | + | + | + | - | - | + |
| Salviani C, 2021 [30] | - | - | + | + | + | + | - | - |
| *Benucci M, 2021 [19] | - | + | + | + | + | + | - | - |
| Terracina KA, 2021 [20] | - | + | + | + | - | + | + | + |
| Watad A, 2021 [52] | + | + | + | + | + | - | + | + |
| Ammitzbøll C, 2021 [21] | + | + | + | + | - | + | - | + |
| Machado PM, 2021 [22] | + | - | + | + | - | + | + | - |
| Bixio R, 2021 [23] | - | + | + | + | - | + | + | - |
| Bartels LE, 2021 [24] | + | + | - | + | - | + | - | + |
| Spiera R, 2021 [25] | - | + | - | + | + | + | - | - |
| Boyarsky BJ, 2021 [26] | - | + | - | + | - | - | - | - |
| Sattui SE, 2021 [27] | - | + | + | + | + | - | + | - |
| Prendecki M, 2021 [28] | + | + | + | - | + | + | + | + |
| Braun-Moscovici Y, 2021 [29] | + | + | + | - | + | + | + | + |

References:

Wells G, Shea B, O’Connell D, et al; The Newcastle-Ottawa scale (NOS) for assessing the quality of nonrandomized studies in meta-analysis. Ottawa, Ontario: The Ottawa Health Research Institute, 2011.

Murad MH, Sultan S, Haffar S, et alMethodological quality and synthesis of case series and case reports. BMJ Evidence-Based Medicine 2018;23:60-63.

Luchini C, Stubbs B, Solmi M, Veronese N. Assessing the quality of studies in meta-analyses: Advantages and limitations of the Newcastle Ottawa Scale. World J Meta-Anal 2017; 5(4): 80-84.
